# How Do You Triage Abdominal Pain in a Patient With Ovarian Cancer?

**DOI:** 10.6004/jadpro.2014.5.5.10

**Published:** 2014-09-01

**Authors:** Paula Anastasia

**Affiliations:** Cedars-Sinai Medical Center, Los Angeles, California

## History

B.V. is a 53-year-old nulliparous woman who was initially diagnosed with recurrent stage 3C epithelial ovarian carcinoma in 2011. She underwent a hysterectomy, bilateral salpingo-oophorectomy, and optimal tumor debulking. Adjuvant therapy consisted of a clinical trial (Gynecologic Oncology Group [GOG] 262) protocol of 6 cycles of IV carboplatin, paclitaxel, and bevacizumab (Avastin) followed by bevacizumab every 3 weeks.

In 2013, B.V. was diagnosed with invasive ductal breast cancer, which necessitated discontinuation of bevacizumab after 12 months of maintenance therapy. She underwent lumpectomy and adjuvant radiation therapy, which was completed in January 2014. Her breast tumor was estrogen-/progesterone-receptor (ER/PR) positive. She was started on tamoxifen.

In February 2014, B.V. had a recurrence of her ovarian cancer. As this was 16 months after she had completed platinum-based chemotherapy for the ovarian cancer, she was considered to have recurrent platinum-sensitive (PS) ovarian cancer. PET/CT scan showed disease in the peritoneal lymph nodes and adjacent to the liver. B.V. tested negative for the BRCA mutation. Because PS patients usually respond to platinum-based therapy when rechallenged (NCCN, 2014), she began IV carboplatin and liposomal doxorubicin (CD) every 28 days (NCCN, 2014).

## Chief Complaint

Two weeks after her fourth cycle of CD, while she was on a weeklong vacation 3,000 miles away from home, B.V. called her advanced practice nurse (APN). She reported that she had woken up at 3 am with severe abdominal pain, estimated at level 8, and had two episodes of emesis. She believed this was a result of the lobster she had had for dinner. She took one hydrocodone/acetaminophen tablet, which decreased the cramps to a level 3 pain score. Although she had intermittent cramping, B.V. was able to return to sleep.

B.V. stated that she had moved her bowels in the morning with the assistance of a stool softener and senna. She denied hard stool and constipation, although she noted that she never feels her stool has been evacuated completely. She did not eat or drink anything that morning for fear of nausea. B.V. reported persistent abdominal cramps, no abdominal distension, and no further emesis.

Her APN instructed her to increase her fluid intake and to resume a bland, soft diet when she became hungry. She had ondansetron on hand; she took 1 tablet in the morning and was instructed to take 1 every 8 hours as needed. The APN reassured B.V., and they discussed some possible etiologies: bowel obstruction vs. delayed chemotherapy-induced nausea vs. food poisoning from her recent shellfish meal.

B.V. was instructed to call her APN if the symptoms worsened. She was told to go to a local urgent care center where she was vacationing if the situation became emergent. B.V. stayed in touch with her APN over the next 24 hours, during which time she described intermittent abdominal pain (level 7/8) with mild nausea and vomiting. She stated that she had had no solid food in the past 24 hours but was tolerating some liquids. B.V. continued to pass gas but did not have a bowel movement. She said her abdomen was soft, not firm or distended. She continued to take pain medication and her antiemetic as needed.

On day 2, B.V.’s friend called the APN to report that B.V. was looking weak and sickly and that she had had persistent vomiting during the night. Her abdominal pain was described as stabbing and persistent. The APN recommended that B.V. be taken to the local hospital to have laboratory tests, possible IV hydration, and an abdominal x-ray.

## Physical Exam and Diagnostic Studies

When B.V. presented to her local urgent care center, her vital signs were normal except for a heart rate of 117. Her body mass index was 33.3 kg/m². Laboratory results included a white blood cell (WBC) count of 0.9 × 1,000/ìL, hemoglobin of 6.7 g/dL, hematocrit of 19.7%, platelet count of 53 x 1,000/ìL, potassium level of 3.1 mEq/L, sodium level of 137 mEq/L, creatinine level of 0.89 mg/dL, and blood urea nitrogen level of 19 mg/dL. An upright and supine abdominal film showed dilated loops of bowel with air fluid levels (see Figure).

**Figure 1:**
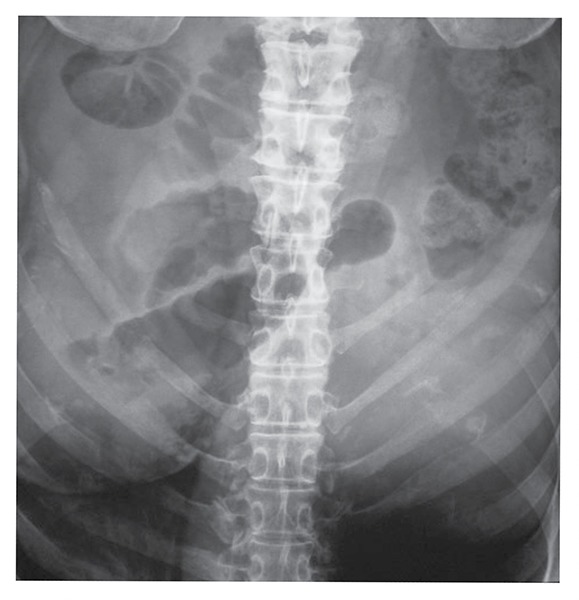
Diagnostic Snapshot

**Figure 2:**
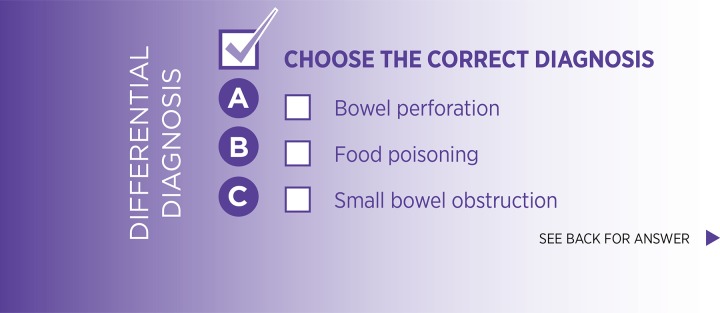
Choose the Correct Diagnosis

## Correct Answer: C

**Small bowel obstruction** (SBO) is a common risk factor for women with a history of surgery and ovarian cancer. Patients often present with an acute onset of abdominal pain, nausea and vomiting, abdominal distension, and an inability to pass gas or stool. An intestinal blockage will cause dilation of the intestine proximal to the blockage, and a distal blockage will cause decompression of the bowel (Soriano & Davis, 2011).

Bowel obstruction can be partial or complete. A complete obstruction is blockage in two locations of the bowel. On physical exam of an acute bowel obstruction, bowel sounds may be high-pitched and associated with pain. As the bowel becomes more distended, sounds will be muffled or absent. Percussion may initially be tympanic and resonant but may become duller as loops of bowel become dilated (Jackson & Raiji, 2011).

Manifestations of dehydration and electrolyte imbalance due to emesis may be present. Fever may represent infection, ischemia, or peritonitis from perforation. B.V. did not manifest the complete constipation or absence of flatulence commonly seen with the associated symptoms of SBO, which is one reason the APN did not immediately suspect it.

## Explanation of Incorrect Answers

**Bowel perforation** is a potentially life-threatening adverse event that may occur in women who receive bevacizumab for ovarian cancer. The incidence generally occurs during administration or immediately after completion of bevacizumab, especially in women with intra-abdominal tumors. In the GOG 218 clinical trial, similar to the chemotherapy and bevacizumab trial that B.V. was enrolled in, 20 of 587 patients (40%) had confirmed bowel perforation during their chemotherapy and bevacizumab therapy (Burger et al., 2014). Presentation of bowel perforation includes fever, guarded severe abdominal pain, and nausea and vomiting. A CT scan will show the location of the perforation, and the WBC count is often elevated due to potential peritoneal infection. As B.V. did not manifest symptoms of fever or acute abdominal pain, the probability of bowel perforation was low.

**Food poisoning**, often referred to as stomach flu, is defined as an infection or illness caused by ingesting contaminated food or water (Gamarra et al., 2013). B.V. was out of town and admitted to eating lobster and seafood, which is a risk factor—if it is raw or undercooked—for someone undergoing chemotherapy. As she had no fever or blood in her in stool, no further workup was done to confirm a foodborne illness.

Although delayed chemotherapy-induced nausea and vomiting was part of the differential diagnosis, the abdominal pain was more worrisome for blockage or infection. B.V.’s laboratory results showed pancytopenia, an incidental finding upon her visit to urgent care. As the abdominal series showed dilated loops of bowel, which was likely the cause of her abdominal pain and gastrointestinal symptoms, there was a low index of suspicion for food poisoning.

## Management

Plain radiography of the abdomen has limited specificity and sensitivity in detecting SBO (Thompson et al., 2007). However, as it is the most cost-effective and quickest test to obtain, it is useful in assessing constipation and potential causes of symptoms (Soriano & Davis, 2011). It is often used before a CT scan for diagnostic and financial reasons. Findings on an abdominal series will show two views: supine and upright. B.V.’s upright view, pictured in the Figure, shows air fluid with distended loops of small bowel. A CT scan can reveal a mass at the site of obstruction, luminal diameter, or irregular thickening of the bowel wall (Jackson & Raiji, 2011). The site or cause of obstruction is best seen on an abdominal CT (not shown), which for B.V. confirmed a high-grade bowel obstruction with fluid-filled and distended loops of small bowel. There was also an associated closed-loop obstruction but no dominant mass or serosal pathology to suggest a malignant obstruction. The etiology for B.V.’s SBO was likely adhesions from her previous surgery. If there had been any sign of possible tumor recurrence, surgical intervention might have been warranted (Soriano & Davis, 2011).

## Follow-up

B.V. was admitted to the hospital overnight for conservative management and placed on NPO (nothing by mouth) status. She received IV hydration and electrolyte replacement. In addition, she received 3 units of packed red blood cells for her anemia and filgrastim (Neupogen) for chemotherapy-induced neutropenia. A nasogastric tube was not inserted, yet it was available should emesis continue.

B.V. began to feel better after pain medication, antiemetics, and IV hydration. She was discharged within 48 hours and given instructions to take in clear liquids, with slow advancement to a low-residue diet. She was restricted from air travel for 1 week due the potential for in-flight bowel dilation (Adusumilli, 2002). B.V. resumed her stool softener and senna and saw gradual normalization of bowel function. She was also being followed with laboratory assessment for her pancytopenia, which was not related to SBO.

The challenge in this scenario was not being able to assess B.V. in the clinic (as she was on vacation in a small town) and having to evaluate her symptoms over the phone. Her radiology films were sent via text and email to the APN and the gynecologic oncologist to assist the local physician in triaging her situation. The urgent care physician wanted to perform surgery on B.V. based on the abdominal radiology reports. Fortunately, after the CT scan showed no immediate danger such as bowel strangulation or perforation, this was not necessary. The APN and the attending physician were able to talk to the local urgent care physician and provide past medical information and recommendations for conservative management.

B.V. returned home and experienced a 1-week delay in her monthly chemotherapy. When she returned home, her CA-125 tumor marker level had increased to 60 ì/mL from 15 ì/mL (normal, < 35 ì/mL). She was reassured that the resolving SBO and airline travel could possibly cause inflammation and a false-positive result or elevation in her tumor marker. A month later, her CA-125 level had returned to 12 ì/mL, which reassured her that she was responding to her chemotherapy.
